# Brain metastases treated with hypofractionated stereotactic radiotherapy: 8 years experience after Cyberknife installation

**DOI:** 10.1186/s13014-020-01517-3

**Published:** 2020-04-17

**Authors:** Laurence Mengue, Aurélie Bertaut, Louise Ngo Mbus, Mélanie Doré, Myriam Ayadi, Karen Clément-Colmou, Line Claude, Christian Carrie, Cécile Laude, Ronan Tanguy, Julie Blanc, Marie-Pierre Sunyach

**Affiliations:** 1 Department of Radiotherapy, Léon Bérard Cancer Center, Lyon, France; 2 Methodology and Biostatistics Unit, Centre Georges François Leclerc, Dijon, France; 3 Department of Medecine, Hôpital d’Aurillac, Aurillac, France; 4 Department of Radiation Oncology, Institut de Cancérologie de l’Ouest, Nantes, France

**Keywords:** Hypofractionated stereotactic radiotherapy, Brain metastases, Radionecrosis

## Abstract

**Background:**

Hypofractionated stereotactic radiotherapy (HFSRT) is indicated for large brain metastases (BM) or proximity to critical organs (brainstem, chiasm, optic nerves, hippocampus). The primary aim of this study was to assess factors influencing BM local control after HFSRT.

Then the effect of surgery plus HFSRT was compared with exclusive HFSRT on oncologic outcomes, including overall survival.

**Materials and methods:**

Retrospective study conducted in Léon Bérard Cancer Center, included patients over 18 years-old with BM, secondary to a tumor proven by histology and treated by HFSRT alone or after surgery. Three different dose-fractionation schedules were compared: 27 Gy (3 × 9 Gy), 30 Gy (5 × 6 Gy) and 35 Gy (5 × 7 Gy), prescribed on isodose 80%. Primary endpoint were local control (LC). Secondary endpoints were overall survival (OS) and radionecrosis (RN) rate.

**Results:**

A total of 389 patients and 400 BM with regular MRI follow-up were analyzed. There was no statistical difference between the different dose-fractionations. On multivariate analysis, surgery (*p* = 0.049) and size (< 2.5 cm) (*p* = 0.01) were independent factors improving LC. The 12 months LC was 87.02% in the group Surgery plus HFSRT group vs 73.53% at 12 months in the group HFSRT. OS was 61.43% at 12 months in the group Surgery plus HFSRT group vs 50.13% at 12 months in the group HFSRT (*p* < 0.0085). Prior surgery (OR = 1.86; *p* = 0.0028) and sex (OR = 1.4; *p* = 0.0139) control of primary tumor (OR = 0.671, *p* = 0.0069) and KPS < 70 (OR = 0.769, *p* = 0.0094) were independently predictive of OS. The RN rate was 5% and all patients concerned were symptomatic.

**Conclusions:**

This study suggests that HFSRT is an efficient and well-tolerated treatment. The optimal dose-fractionation remains difficult to determine. Smaller size and surgery are correlated to LC. These results evidence the importance of surgery for larger BM (> 2.5 cm) with a poorer prognosis. Multidisciplinary committees and prospective studies are necessary to validate these observations.

## Background

Brain metastases (BM) occur in up to 20–40% of all cancer patients [1]. Their incidence increases with the development of performant imaging and the advances of systemic therapies providing a longer survival [2]. Neurologic symptoms associated to BM can significantly impact quality of life [3]. Local treatment arsenal for BM includes neurosurgery, whole brain radiotherapy (WBRT), stereotactic radiosurgery (SRS) and more recently hypofractionated stereotactic radiotherapy (HFSRT) [4]. SRS is largely reported in literature. The use of SRS is however correlated to a high risk of radionecrosis [5]. Large lesions distant from critical structures are preferably treated by surgery [6]. In case of surgical contraindication HFSRT may be an option [7]. HFSRT provides radiobiological advantages compared to SRS when BM are larger or closer to critical structures such as brainstem or chiasm [8, 9]. HFSRT offers the ability to deliver higher dose in terms of radiobiological equivalent dose on large BM, and to reduce toxicity of normal brain tissue [9]. HFSRT probably improves the therapeutic ratio and increases tumor control probability as suggested by an in silico study [8]. However, one of HFSRT relevant challenges is to assess the optimal dose and fractionation regimen associated to local control of BM, according to size and volume [9].

The objectives of this study were to investigate prognostic factors influencing BM local control (LC), overall survival (OS) and radionecrosis (RN) development, in patients treated by HFSRT in the clinical practice of a single institution over 8 years. Thus, the effect of surgery plus HFSRT was compared with exclusive HFSRT on oncologic outcomes, including overall survival.

## Materials and methods

### Study population

This is a retrospective and monocentric study conducted at the Léon Bérard Cancer Center in Lyon. Eligible patients were over 18 years of age, treated by exclusive intracranial HFSRT or surgery with post-operative HFSRT. All patients had a histological proof of primary tumor by biopsy. Patients who underwent prior WBRT or surgery were also included in outcome analysis. Exclusion criteria were the presence of primary intracranial tumors (e.g. meningioma or glioblastoma), and other stereotactic irradiation. This study was ethically approved by the Léon Bérard Cancer Center institutional board.

### Data collection

The main clinical data were extracted from computerized patients’ records were: age, sex, Karnofsky performance status (KPS), primary tumor histology, extracranial disease control. Prognostic scores Recursive Partitioning Analysis (RPA) [10], modified Recursive Partitioning Analysis (mRPA) [11, 12], Graded Prognostic Assessment (GPA) [13], Diagnostic-Specific Graded Prognostic Assessment (DS-GPA) were calculated for each patient [13] . Neurological symptoms prior BM treatment (motor or sensitive dysfunction, intracranial hypertension, and aphasia) were also reported from retrospective data [3]. The assessment of neurological symptoms was only qualitative. We also took into account the systemic treatments delivered concomitantly or within 1 month of radiotherapy, as well as whether or not the extracerebral disease was controlled. The systemic treatments included chemotherapy, targeted therapy and immune-checkpoint inhibitors [14] .The fact that the extracerebral disease is not controlled may be related either to a bifocal recurrence or to an initial metastatic tumour.

Regarding BM characteristics, reported informations were maximal diameter, number and location. BM diagnosis was performed either on a cerebral MRI, biopsy or operative piece histology. Percentage of meningial spreading was also assessed for brain metastases treated by surgery and HFSRT [15] .

### Hypofractionated stereotactic radiotherapy (HFSRT)

For each patient, surgery or HFSRT indication was discussed at a multidisciplinary staff meeting. HFSRT was delivered by Cyberknife™ (Accuray Inc., Sunnyvale, CA). The non-injected simulation CT-scanner was performed in supine position.

A thermoformed frame mask system was used. MRI images (1 mm slice thickness) were registered with planning CT scan (1 mm slice thickness) on Multiplan (Accuray) workstation. Gross Tumor Volume (GTV) corresponded to contrast-enhancing lesion on T1 sequences of brain fusion MRI. If an adjuvant HFSRT was performed, GTV represented the surgical cavity. In both cases, a 2 mm margin was systematically added to GTV to generate the planning target volume (PTV). When BM was closer to critical structures, margin could be reduced to 0–1 mm and organs at risk (OAR) was excluded from PTV.

The main risk-delineated organs were brainstem, optic nerves, chiasm and hippocampus. Dose and fractionation regimens were chosen at the discretion of the radiotherapist, depending on the BM size, OAR proximity (brainstem, corpus callosum, optic nerves and chiasm), and prior WBRT or surgery. For the smaller lesions, 3 fraction protocols are frequently used. Concerning larger lesions or lesions located near OAR or in functional sites, 5 fraction schedules are used. The prescription isodose was 80%. Treatment plan validation was done according to PTV coverage (up to 95%) and respect of maximal dose received by OAR according Timmerman recommendations [16].

Brain metastases or post-operative cavities were irradiated on alternate day, every other day.

### Follow-up after HFSRT

The follow-up was based on a clinical neurological examination and on a brain MRI every 3 months 1st year and every 6 months the following years, according to ANOCEF recommendations [17].

### Study endpoints

Primary endpoint was local control defined by stability, partial or complete response of the lesion after HFSRT. Secondary endpoints were overall survival (OS) and rate of radionecrosis (RN). OS corresponded to the interval between the date of HFSRT and date of last follow-up or death. The definition of RN was more extensive and heterogeneous. The RN diagnosis was performed on MRI surveillance or histology [18] . Further images as spectrometric MRI could be requested, if there was uncertainty between RN or local failure diagnosis [19, 20]. MRI were reviewed by an experimented radiotherapist. Stability of BM size over several months was in favor of RN.

### Statistical analysis

Categorical variables were presented as numbers (percentage) and continuous variables were described as median and standard deviation (minimum and maximum values).

LC and RN analyses were performed using a per-lesion basis. Evaluation of OS was conducted on per-patient basis. Kaplan Meier curves were generated to estimate both endpoints. Log-Rank test was used to assess predictive factors on survival outcomes.

For OS and LC estimates, living patients were censored at the last follow-up and for others, at the most recent visit or death.

Cox univariate and multivariate regression were performed to determine independent predictive factors of local control and OS. Co-variables with *p*-value less than 0.2 on univariate analysis, were introduced in the statistical model. Variables with more than 20% missing data were not included in the multivariate model. All tests were two sided and *P* values were considered significant when less than 0.05. Analyzes were performed using the SAS 9.4 statistical software.

## Results

### Patient population

Between January 2011 and January 2018, a total of 427 patients were treated by HFSRT at Léon Bérard Cancer Center. Median age was 62 years-old (18–87). There were 188 men and 239 women. WBRT was performed before HFSRT on 39 patients (9%).

Patients characteristics are presented in Table 1. Patients were divided into two groups Surgery plus HFSRT and HFSRT. Both groups were similar in terms of age, sex, GPA, DS-GPA, systemic treatment and control of primary tumor. Significant differences between both groups were based on extracranial control, neurologic symptoms, RPA and mRPA.

**Table 1:** Characteristics of patients

	Surgery + HFSRT ***N*** = 99	HFSRT ***N*** = 328	pValue
**Age**			0.1511
Median [min - max]	59.0 [13.2–87.0]	60.8 [8.3–85.9]	
**Sex**			0.4587
male	52 (52.5%)	185 (56.7%)	
Female	47 (47.5%)	143 (43.3%)	
**KPS**			0.25
100–90	38 (38%)	96 (30%)	
80–70	46 (46%)	169 (52%)	
< 70	15 (15%)	60 (18%)	
**RPA**			**<.0001**
1	29 (29.3%)	35 (10.7%)	
2	55 (55.6%)	233 (71.0%)	
3	15 (15.2%)	60 (18.3%)	
**m RPA**			**< 0.001**
1 + 2a	55 (56%)	89 (27%)	
2b	17 (17%)	90 (28%)	
2c + 3	27 (27%)	146 (45%)	
**GPA**			0.0868
< = 3	98 (99.0%)	311 (94.8%)	
> 3	1 (1.0%)	17 (5.2%)	
**DS-GPA**			0.2485
< = 3	78 (87.6%)	265 (91.7%)	
> 3	11 (12.4%)	24 (8.3%)	
Not applicable	10	39	
**Survival at the last follow-up**			**0.0227**
Alive	63 (63.6%)	166 (50.6%)	
Dead	36 (36.4%)	162 (49.4%)	
**Extracranial control**			**0.0001**
Yes	49 (49.5%)	116 (34.4%)	
No	41 (41.4%)	182 (55.5%)	
NC	9 (9.1%)	30 (9.1%)	
**Neurologic symptoms**			**<.0001**
No	77 (77.8%)	133 (52.0%)	
Yes	22 (22.2%)	123 (48.0%)	
NC	0	72	
**Systemic treatement**			0.3197
No	56 (58.3%)	197 (64.0%)	
Yes	40 (41.7%)	111 (36.0%)	
**Control of primary tumor**			0.6333
Yes	49 (50.5%)	148 (47.7%)	
No	50 (49.5%)	180 (52.3%)	

### Brain metastasis

A total of 535 BM was identified in the study and 400 BM (389 patients) were followed with an MRI. Median size was 2.3 cm (0.6–6.5 cm). The main histology and location was lung cancer (55%), followed breast cancer (12%), clear cell renal carcinoma (10%) and melanoma (5.2%). On 535 BM, 88 (20%) had surgical resection before HFSRT. Among BM treated by surgery and HFSRT, 13 cases of meningial spreading were detected on MRI or cytology. BM were split into radio resistant group (clear cell carcinoma cancer, sarcoma and melanoma) and radiosensitive group (other histological types), counting 120 and 414 BM respectively.

In the group of Surgery plus HFSRT (Table 2), 71% BM were larger than 2.5 cm vs 36.7% in comparison with the group HFSRT (*p* < .0001). More than half of post-operative cavities received a dose of 30 Gy in 5 fraction or 35 in 5 fractions. In the group HFSRT, a dose of 27 Gy in 3 fractions was applied on 47.1% (*n* = 202). Concerning prior systemic treatment, there was no significant differences between both groups (Table 1).

**Table 2:** Characteristic of treated brain metastasis

	Surgery + HFSRT ***N*** = 105	HFSRT ***N*** = 429	pvalue
**Size**			**<.0001**
< 2.5 cm	29 (29.0%)	246 (63.2%)	
2.5–4 cm	47 (47.0%)	123 (31.6%)	
> 4 cm	24 (24.0%)	20 (5.1%)	
	5	40	
**Total dose and fractionation (gy)**			**<.0001**
27	12 (11.4%)	202 (47.1%)	
30	54 (51.4%)	108 (25.2%)	
35	39 (37.1%)	119 (27.7%)	
**WBRT**			0.0329
Yes	3 (2.9%)	35 (8.1%)	
No	102 (97.1%)	394 (91.9%)	
**Tumor Histology**			0.5626
Breast	13 (12.4%)	53 (12.4%)	
Kidney	7 (6.7%)	40 (9.3%)	
Lung	64 (61.0%)	231 (53.8%)	
Melanoma	3 (2.9%)	25 (5.8%)	
Other	18 (17.1%)	80 (18.6%)	
**Location**			0.0614
Brainstem	0 (0.0%)	15 (3.5%)	
Infratentorial	32 (30.5%)	100 (23.3%)	
Supratentorial	73 (69.5%)	314 (73.2%)	
**radiosensible**			0.4984
Yes	84 (80.0%)	330 (76.9%)	
No	21 (20.0%)	99 (23.1%)	

### Local control

**Fig. 1:**
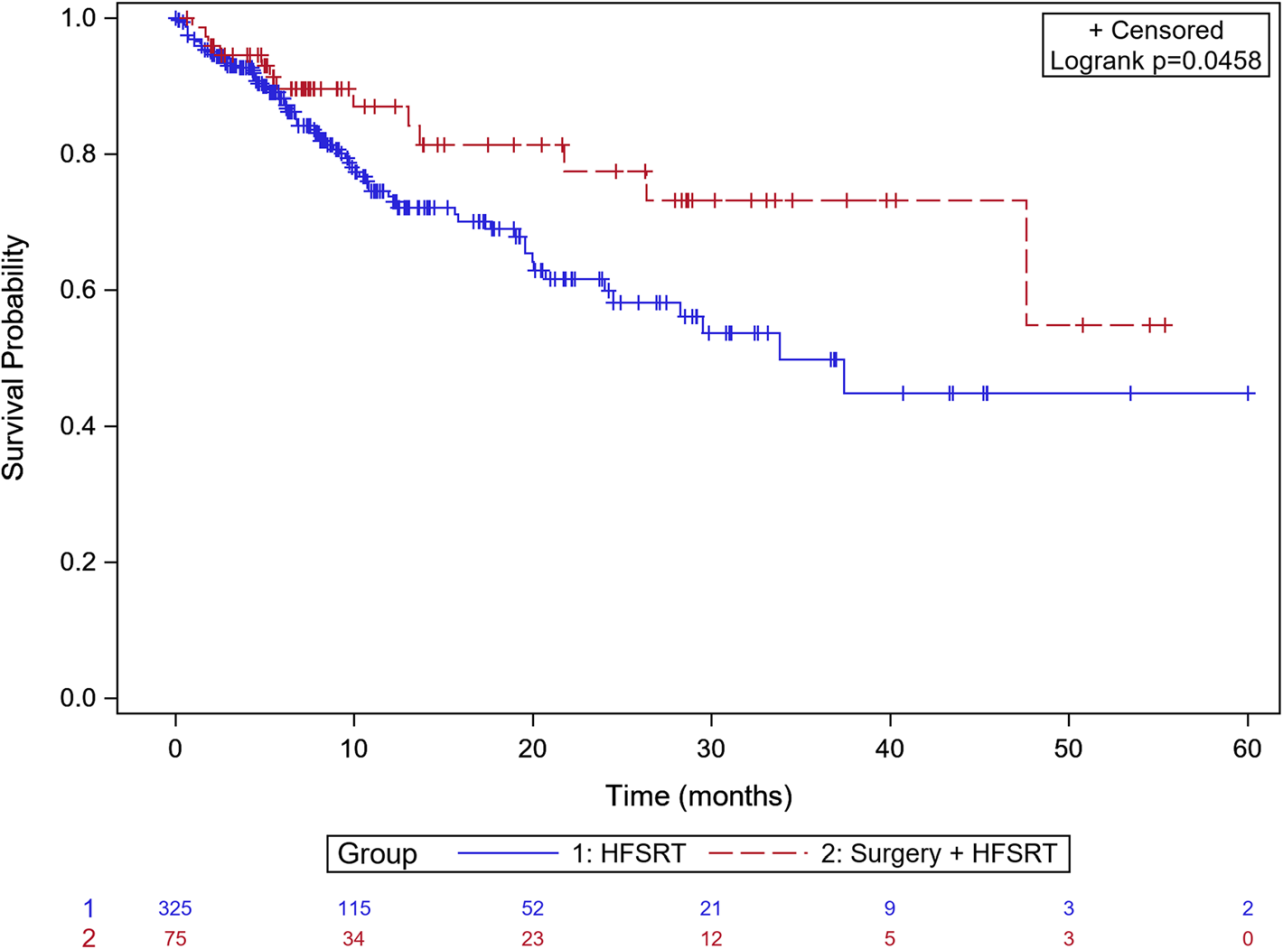
Kaplan Meier curve of local control

**Fig. 2:**
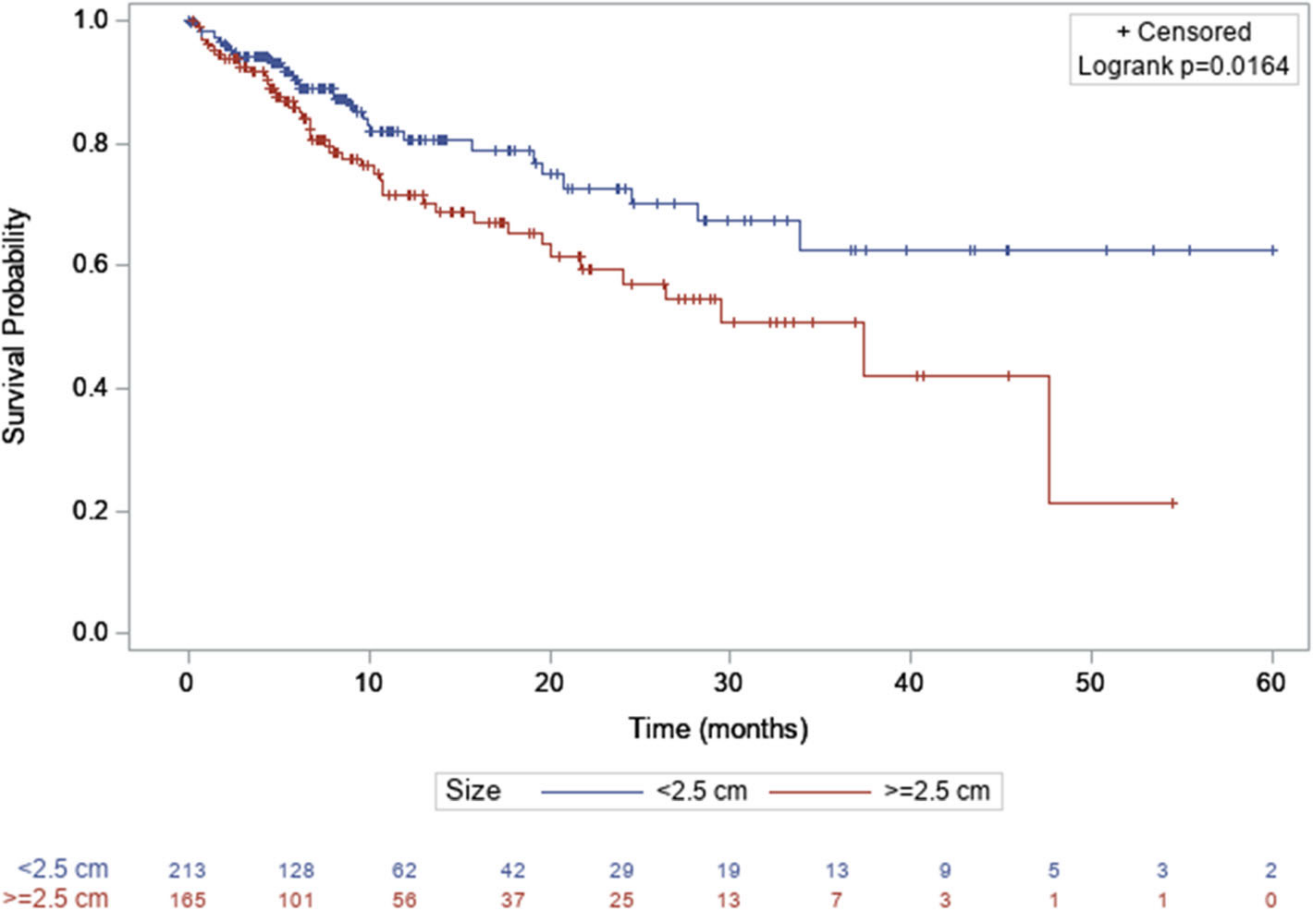
Kaplan Meier curve of local control in function brain metastases size

Analyses were performed on 400 BM with available follow-up images. Patients excluded from analyses of LC because of inadequate imaging follow up were not statistically different from patients included in analyses.The median follow-up is 40 months (1–60 months). If we consider the whole group, local control at 6 months, 1-year and 2-years was 88%, 76. 5 and 63.9% respectively. In the group of patients treated with Surgery +HFSRT, the local control was 89.58% at 6 months, 87.02% at 12 months 77.53% at 24 months vs 88.17% at 6 months, 73.75% at 12 months, 59.93% at 24 months in the groupe of patients treated with exclusive HFSRT (Fig. 1). LC was improved when size was less than to 2.5 cm (*p* = 0.0164) (Fig. 2). On univariate analyses and multivariate, maximal BM size (< 2.5 cm) and prior surgery were the only predictive factor of LC (Table 4). Systemic treatment and fractionation schedules were not statically associated with LC (Table 4).

**Fig. 3:**
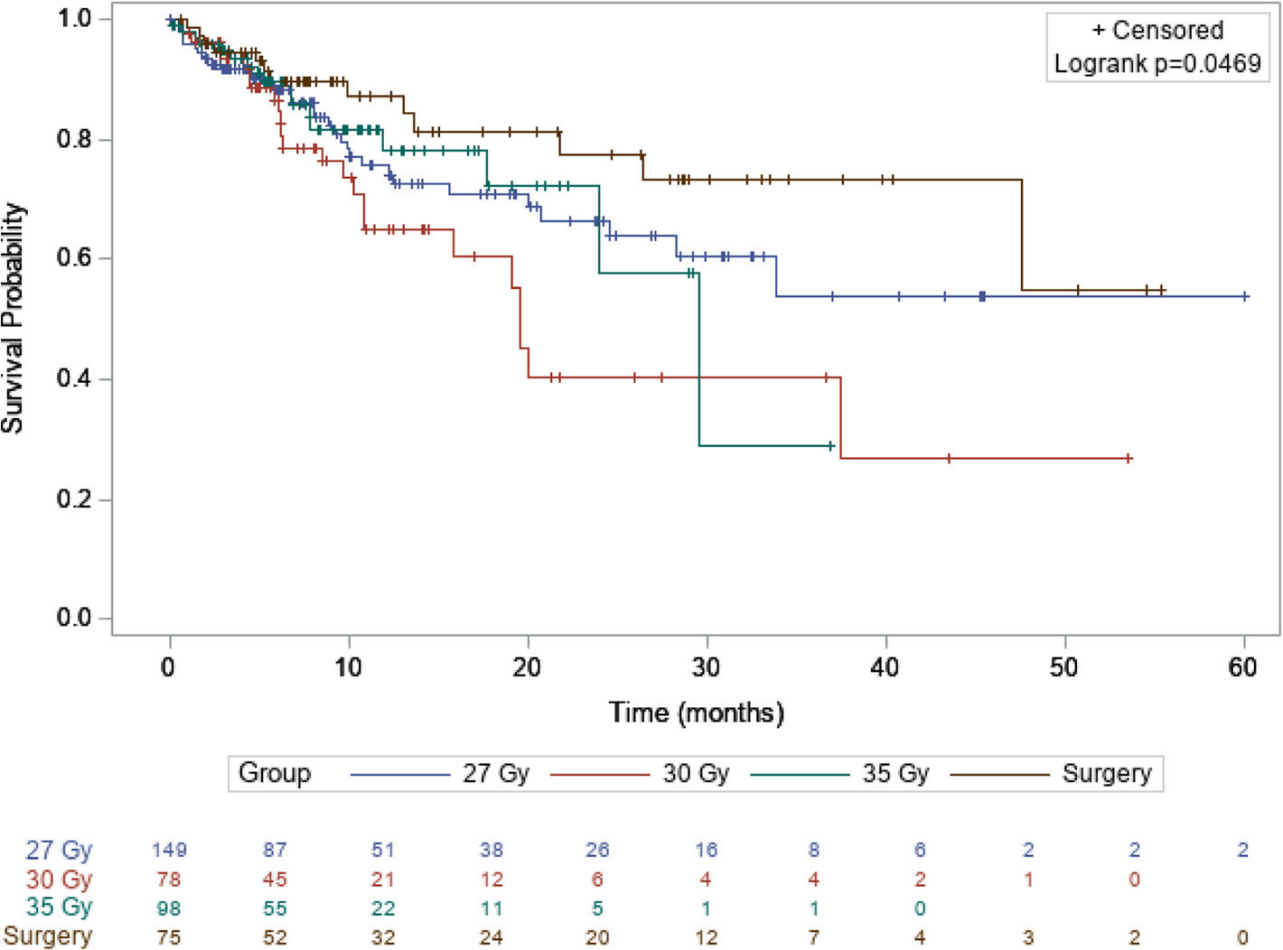
Kaplan Meier curve of local control according to surgery and dose-fraction regimens. The group surgery represents all patients treated by surgery followed by HFSRT (27, 30 or 35 Gy)

For further analyses, BM were divided into 4 groups of treatment modality. The group of patients treated by surgery plus adjuvant HFSRT was compared to 3 other groups of BM treated by 3x9Gy, 5x6Gy, and 5x7Gy schedules (Fig. 3). Surgery had a significant impact on LC (*p* = 0.0469), whereas dose-fraction regimen had no impact (Table 3).

**Table 3:** Univariate and multivariate analyses on local control (LC)

Univariate analyses					Multivariate analyses			
Variable	N	HR	IC95%	p	HR	IC95%		p
**Size**	*N* = 378			**0.0178**				**0,0072**
> =2.5 cm vs < 2.5 cm		1.699	[1.096–2.634]		1.909	[1.191–3.060]		
**Fractionation**	*N* = 400			0.3902				0,2072
30 Gy in 5 vs 27 Gy in 3		1.331	[0.823–2.151]		0.917	[0.508–1.655]		
35 Gy in 5 vs 27 Gy in 3		0.955	[0.553–1.649]		1.453	[0.853–2.477]		
**Control of primary tumor**	*N* = 383			0.0598				
Yes vs No		0.661	[0.429–1.017]					
**Systemic treatment**	*N* = 382			0.9556				
Yes vs No		0.987	[0.630–1.546]					
**Surgery**	N = 400			**0.0490**				**0,0044**
Yes vs no		1.816	[1.002–3.289]		2.506	[1.333–4.712]		
**Group**	N = 400			**0.0490**				
Surgery + HFSRT vs HFSRT		0.551	[0.304–0.998]		0.437	[0.235–0.812]		**0,0088**

### Overall survival

**Fig. 4:**
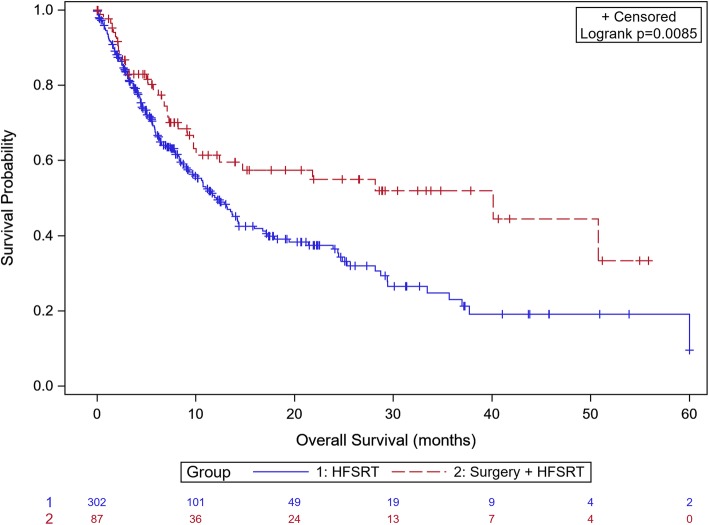
Kaplan Meier curve of overall survival according to surgery

With a median follow-up of 40 months (1–60 months) median overall survival was 13.17 months (10.68–17.22). One-year OS was 52.7%. Overall survival at 12 months was 61.43% in the surgery +HFSRT group vs 50.13% in the HFSRT group (*p* < 0.0085) (Fig. 4).

On Cox univariate model, clinical parameters such as sex, RPA, mRPA, GPA, DS-GPA, control of extra cerebral tumor, prior surgery and KPS were significantly associated with overall survival, whereas age, DS-GPA, radio sensitivity were not (Table 4). In Cox Multivariate model, prior surgery (OR = 1.86; *p* = 0.0028), sex (OR = 1.4; *p* = 0.0139), control of extra cerebral tumor (OR = 0.671, *p* = 0.0069) and KPS < 70 (OR = 0.769, *p* = 0.0094) were independently predictive for OS (Table 4).

**Table 4:** Univariate and multivariate analysis on OS

		Univariate analyses			Multvariate analyses		
Variable	N	HR	IC95%	p	HR	IC95%	p
**Age**	*N* = 389			0.0891			0.4633
**< 70 vs ≥ 70**		1.402	[0.69–1.525]		1.473	[0.574–1.353]	
**Sex**	*N* = 387			**0.0024**			**0.0139**
**Female vs Male**		1.578	[1.175–2.12]		1.578	[1.08–2.00]	
**KPS**	N = 389			**0.0094**			**0.0094**
v < 70 vs ≥70		0.622	[0.435–0.89]		0.769	[0.522–1.133]	
**RPA**	N = 389			**0.0301**			0.4
**II-III vs I**		1.756	[1.08–2.854]		0.799	[0.472; 1.35]	
**m RPA**	N = 389	1.700		**0.001**	1.74	[1.12–2.69]	**0.014**
**II-III vs I**			[1.1–2.854]				
**GPA**	N = 389			**0.0342**			0.1411
< 3 vs ≥3		0.642	[0.427–0.968]		0.712	[0.45–1.12]	
**DS-GPA**	*N* = 345			0.3525			
< 3 vs ≥3		0.842	[0.587–1.209]				
**Surgery**	N = 389			**0.00111**			**0.0028**
Yes vs No		1.623	[1.117–2.357]		1.86	[1.24–2.79]	
**Symptoms**	*N* = 322			0.696			
No vs Yes		0.956	[0.697–1.312]				
**Single BM**	N = 389			0.696			
Yes vs No		1.074	[0.752–1533]				
**Radiosensitivity**	N = 389			0.2592			
Yes vs No		0.83	[0.601–1.147]				
**Control of primary tumor**	*N* = 373			**0.0036**			**0,0087**
Yes vs No		0.645	[0.480–0.867]		0.671	[0.497–0.904]	
**Systemic treatment**	*N* = 370			0.6987			
Yes vs No		0.942	[0.694–1.277]				
**Group**	N = 389			**0.0092**			**0,0089**
Surgery + HFSRT vs HFSRT		0.601	[0.419–0.884]		0.410	[0.419–0.884]	

### Late toxicity: radionecrosis

With a median follow-up of 16.4 months (5.6–71.8 months), a total of 20 cases (5%) of RN were reported above 400 BM with regular MRI follow-up. All patients presented neurologic symptoms. For RN diagnosis, follow-up MRI was mandatory (at least 2 consecutive MRI with RN diagnosis). Follow-up MRI performed were multi-modal with T1, T2, FLAIR and perfusion sequences. Most of RN occurred on BM originated from lung cancer (*n* = 10; 50%), followed by breast cancer (*n* = 6; 30%), melanoma (*n* = 2; 10%), digestive tract (*n* = 1; 5%), head and neck cancer (*n* = 1; 5%). Median size of BM was 2.3 cm (1–4 cm). RN occurred on 7 post-operative cavities (35%). Two patients needed surgery because of neurologic symptoms.

## Discussion

This study aimed to assess the efficiency and safety of HFSRT with or without surgery. In our institution, HFSRT and SRS were used upfront WBRT, even if multiple BM were observed (> 3) to reduce late neurocognitive toxicity. This practice was supported by EORTC study comparing WBRT vs surveillance after surgery or SRS [21]. Their data demonstrated that median overall survival was not significantly different between both groups (10.7 months vs 10.9 months; *p* = 0.89). More recently a second analysis was performed and showed similar results: Churilla et al. compared limited BM 1 to 3 vs extended BM (> 3) and concluded that number of BM treated by SRS was not associated to overall outcome [22].

Patients with multiple BM were more likely to have extracranial progression. Therefore, we found an estimated overall 1 year survival of 52.7%, which is lower than what it was found in some studies [23] previously reported previous studies.

For each patient RPA, mRPA, GPA, and DS-GPA prognostic scores were calculated and reported. On univariate analyses, RPA mRPA and GPA were significantly associated with overall survival. On multivariate analysis, only mRPA was significantly associated to overall survival. According literature, mRPA is more discriminant than RPA [11]. The group 2 from RPA score is heterogenous [12]. Thus, many studies suggested that a subclassification of group 2 is necessary to predict more accurately overall survival [12]. Our finding might be in agreement with those observations.

However our study might be limited by the fact that nomograms were not systematically used before HFSRT indication. Moreover, even if these scores systems have been validated, they were developed based on patients treated by WBRT and not by SRS or HFSRT alone [13, 24]. Each score refers to different predictive factors such as age, BM number, histology of primitive tumor and performance status score [22, 25]. Nieder et al. suggested that all these criteria can not be reported in some clinical situations [26]. Other publications showed that cumulated volume size and surgery should be integrated in nomogram [7, 27]. Therefore, it is impossible to take into account all those factors highlighting the importance of a multidisciplinary staff meeting.

In the present series local control was 88, 76.5 and 63.9% at 6 months, 1 year and 2 years, respectively. This is in agreement with literature showing that HFSRT is an efficient treatment for BM (Table 5). Those studies are mainly retrospective and included small cohorts. However, their data reported a satisfying local control at 1 year (76 and 93%). The only prospective study based on HFSRT included 51 patients and local control was 76% at 1 year [28].

**Table 5:** Literature review of hypofractionated stereotactic studies

Publication date	Nb pts/BM	Median Volume (cc) or size (mm)	Regimen	Isodose (%)	WBRT (%)	LC at 12 m (%)	Median OS at 12 m (%)	RN (%)	Prognostic factors
Matsuyama et al. 2005–2009	299/573	8.6 mm (2.8–47.4)	Variable regimen	NC	10	94	57,8	2	Size
LIiang-hua et al. 2001–2011	171/354	14.3 cc (0.16–86)	4 × 8 Gy	80–90	68	68	51	6	WBRT for OS
Martens et al. 2006–2010	75/108	1 cc (0.1–29.2)	6Gyx5 6-7x5gy 7-10 × 4 Gy	NC	52	52	35	1	Volume
Giubilei et al. 2001–2006	30/44	4.8 cc (0.4–24.3)	3 × 6 Gy 4 × 8 Gy	Isocenter	100	86	36	0	NC
Marchetti et al. 2001–2005	65/81	8 cc (0.3–48.2)	3 × 8 Gy	80	44	58	25	1	NC
Fahrig et al. 2000–2005	150/228	30 mm	5 × 6-7Gy 10x4Gy 7 × 5 Gy	NC	34	NC	66	NC	Dose volume
Jiang et al. 2003–2009	40/NC	30 mm (3.1–5.5) 17,5 cc (6–64.6)	4 × 10 gy	90	25	94.2	55.3	2.5	Histology KPS
Scorsettiet al. 2004–2007	78/113	3.3 cc (0.1–28)	6x4Gy 7x5Gy 1 × 20 Gy	80	10	69	NC	NC	RPA
Nagai et al. 2009–2013	54/128	1.9 cc (0.1–18)	4 × 7 Gy	80	NC	91	52	0	NC
Fokas et al. 2012	214/214	30 mm	7 × 5 Gy 4x10Gy	Isocenter	0	90	31	NC	RPA
Ernst et al. 2003–2005	51/72	3 cc	5 × 7 Gy 5 × 6 Gy	NC	NC	NC	NC	NC	V4 < 20 cc
Inoue et al. 2010–2014	88/92	10 -74 cc	3 × 9 Gy 3x10Gy	57	0	90	NC	0	V14 RN

*LC* local control, *V14* Irradiated volume receiving 14 Gy, *RN* Radionecrosis, *RPA* Recursive Patitioning Analysis, *m* months, *Vol* Volume, *NC* not communicated, *pts*. patients, *Nb* Number

Our study analyzed dose-fractionation prescribed on the same isodose (80%). Intent was to adapt fractionation regimen to clinical situations. Higher dose-fractionation 5 × 7 Gy corresponded to a biological equivalent dose with α/β = 10 (BED10 Gy) of 59.9 Gy, was delivered on larger BM (> 2.5 cm). Schedules 3 × 9 Gy and 5 × 6 Gy corresponding to lower BED10 Gy of 51.3 Gy and 48 Gy respectively, were more often applied on post-operative cavity and smaller lesions [29]. In Table 2, we can see that physician’s adapted volume fractionation, number of lesions prior to WBRT or surgery. In regard of literature, rare studies assessed the indication of HFSRT for smaller BM (< 2.5 cm) [29, 30]. Studies that analyzed HFSRT outcomes were mainly focused on larger BM [7]. In our study, we also included smaller BM treated by HFSRT at the proximity of eloquent structure. Although, dose and fractionation schedule prescriptions were influenced by clinical parameters, none of the HFSRT fractionation schedules emerged as an optimal treatment leading to a significantly improvement of local control no matter BM size.

In our study BM size was the main prognosis factor influencing local control. Local control was better when BM were smaller < 2.5 cm. Our results are consistent with literature showing that size is a robust prognosis factor of local control upon SRS and HFSRT [8, 27]. However, definition of large BM is heterogeneous among studies, making the comparison difficult [6].

Our data demonstrated that larger BM (> 2.5 cm) had worse local control whatever the dose and fractionation. In Léon Bérard Cancer Center, a dose escalation was performed up to 35 Gy on larger tumors. Despite this dose escalation, this suggests that larger BM still have poorer local control compared to smaller lesions. Adaptation of fractionation to tumor volume failed to compensate the bad prognostic induced by tumor volume. Some authors suggested the use of a further fractionated treatment. Determining optimal dose is a controversial debate [31]. Partly this can be explained by fundamental radiobiology [29]. The larger the BM is, the more important the hypoxic fraction is, leading to radio-resistance [30]. Another explanation may be that dose-fractionation used in brain cannot reach higher BED10 Gy due to dose constraints [16]. In extracranial stereotactic body radiotherapy, it is well established that dose escalation is strongly linked to cell death and tumor decrease when BED10 Gy is higher [23, 32, 33].

Late toxicity (radio necrosis) was limited to 5% in this large cohort. All patients with diagnosed radionecrosis were symptomatic. Radionecrosis diagnosis was performed on MRI follow-up most of the time. Minitti and al. performed analyses on a large cohort including 289 patients [34]. A group treated by SRS was compared to a group treated by HFSRT. Nineteen percent of patients in the SRS group vs 9% in the HFSRT group presented a radionecrosis. In our study this rate was lower. The incidence of radionecrosis depends on the definition. Asymptomatic RN are not reported in this study. Nonetheless, Zindler et al. demonstrated that HFSRT reduces RN rate [8] . Indeed, the higher the fraction number, the more OAR are protected from late toxicity. A 24 studies meta-analyse conducted by lerhar et al. also demonstrated that HFSRT offers lower rate of radio-necrosis with an improved LC [35].

Surgery is a prognostic factor correlated to local control and overall survival on multivariate analysis. This finding suggests that surgery should be proposed for BM larger than 2.5 cm, when this feasible without morbidity. Previous studies compared post-operative SRS versus SRS alone. Local control after surgery alone is estimated to 50% [4]. A recent prospective phase III study compared follow-up or SRS after surgery [36]. The local free recurrence at 1-year was 43% in the group treated by surgery vs 72% the group treated by surgery plus SRS. Lamba et al. found that surgery plus SRS was associated with an improved LC and overall survival in comparison to SRS alone [37].

To our knowledge, this is one of largest studies comparing HFSRT and surgery plus HFSRT. In the EORTC study comparing SRS or surgery with or without WBRT, including 289 patients, local control and global survival were comparable between both groups. This suggested that SRS is as efficient as surgery. For patients not eligible to surgery due to multiple or deep BM, optimal dose-fractionation should be assessed by randomized prospective studies. Association with systemic agents should be further investigated [38–40].

## Conclusion

Our study suggests that HFSRT is an efficient and safe treatment. Lesion size and surgery are correlated to a robust local control. Surgery may provide a better outcome in term of overall survival. This study confirms the importance of surgery in BM management. Further studies are required to assess the interest of dose escalation in BM management.

## Data Availability

Datasets generated for this study are available upon request to the corresponding author.

## Abbreviations

ANOCEF: Association des Neuro-Oncologues d’Expression Française
BED10 Gy: Biological equivalent dose with α/β = 10
BM: Brain metastases
DS-GPA: Diagnostic-Specific Graded Prognostic Assessment
GPA: Graded Prognostic Assessment
GTV: Gross Tumor Volume
HFSRT: Hypofractionated stereotactic radiotherapy
KPS: Karnofsky performance status
LC: Local control
OAR: Organs at risk
OS: Overall survival
PTV: Planning target volume
RN: Radionecrosis
RPA: Recursive Partitioning Analysis
SRS: Stereotactic radiosurgery
WBRT: Whole brain radiotherapy
